# Supplementary material for: Maternal Reassurance, Satisfaction, and Anxiety after First-Trimester Screening for Aneuploidies: Comparison between Contingent Screening and Universal Cell-Free DNA Testing
Source: Diagnostics (Basel). 2024 Jun 6;14(11):1198. doi: 10.3390/diagnostics14111198 (PMC11172334; doi:10.3390/diagnostics14111198)
Supplement: Supplementary file 1 [file diagnostics-14-01198-s001.zip › diagnostics-3012881-supplementary.pdf]

diagnostics

MDPI

Supplermentary material
PRAQ Questionnaire

The PRAQ-R/R2 form as used in FinnBrain (translated back to English), containing both the old item (8) as well as the new replacing item (1). *: New item applicable for all pregnant women regardless of parity. This item replaces **: Old item only applicable for nulliparous women.
Please circle each answer that applies most accurately to your situation.
Answer categories:

1. Absolutely not relevant
2. Hardly ever relevant
3. Sometimes relevant
4. Reasonably relevant
5. Very relevant.

1. I am anxious about the delivery.	1	2	3	4	5
2. I am worried about the pain of contractions and the pain during delivery.	1	2	3	4	5
3. I am worried about the fact that I shall not regain my figure after delivery.	1	2	3	4	5
4. I sometimes think that our child will be in poor health or will be prone to illnesses.	1	2	3	4	5
5. I am concerned about my unattractive appearance.	1	2	3	4	5
6. I am worried about not being able to control myself during labour and fear that I will scream.	1	2	3	4	5
7. I am worried about my enormous weight gain.	1	2	3	4	5
8. I am anxious about the delivery because I have never experienced one before.	1	2	3	4	5
9. I am afraid the baby will be mentally handicapped or will suffer from brain damage.	1	2	3	4	5
10. I am afraid our baby will be stillborn, or will die during or immediately after delivery.	1	2	3	4	5
11. I am afraid that our baby will suffer from a physical defect or worry that something will be physically wrong with the baby.	1	2	3	4	5

Total sum scores PRAQ-R: Items 2–11
Old subscale PRAQ-R Fear of giving birth: Items 2, 6, 8
Total sum scores PRAQ-R2: Items 1–7, 9– 11
New subscale PRAQ-R2 Fear of giving birth: Items 1, 2, 6
Subscale Worries about bearing a handicapped child: Items 4, 9–11
Subscale Concern about own appearance: Items 3, 5, 7

STAI questionnaire

A number of statements which people have used to describe themselves are given below. Read each statement and then circle the most appropriate number to the right the statement to indicate how you feel right now, at this moment. There are no right or wrong answers. Do not spend too much time on any one statement but give the answer wbich seems to describe your present feelings best.

	Not at all	Somewhat.	Moderately	Very much
1. I feel calm	1	2	3	4
2. I am tense	1	2	3	4
3. I feel upset	1	2	3	4
4. I am relaxed	1	2	3	4
5. I feel content	1	2	3	4
6. I am worried	1	2	3	4

Please make sure that you have answered *all* the questions.
